# Recoding of Nonsense Mutation as a Pharmacological Strategy

**DOI:** 10.3390/biomedicines11030659

**Published:** 2023-02-22

**Authors:** Gazmend Temaj, Pelin Telkoparan-Akillilar, Nexhibe Nuhii, Silvia Chichiarelli, Sarmistha Saha, Luciano Saso

**Affiliations:** 1 Faculty of Pharmacy, College UBT, 10000 Prishtina, Kosovo; 2 Department of Medical Biology, Faculty of Medicine, Yuksek Ihtisas University, 6530 Ankara, Turkey; 3 Department of Pharmacy, Faculty of Medical Sciences, State University of Tetovo, 1200 Tetovo, North Macedonia; 4 Department of Biochemical Sciences “A. Rossi-Fanelli”, Sapienza University of Rome, 00185 Rome, Italy; 5 Department of Biotechnology, GLA University, Mathura 00185, Uttar Pradesh, India; 6 Department of Cardiovascular, Endocrine-Metabolic Diseases, and Aging, Italian National Institute of Health, 00161 Rome, Italy; 7 Department of Physiology and Pharmacology “Vittorio Erspamer”, Sapienza University of Rome, 00185 Rome, Italy

**Keywords:** nonsense-mediated decay, SURF complex, drug inhibition, aminoglycoside, non-aminoglycosides, pharmacological perspective, pharmacological therapy

## Abstract

Approximately 11% of genetic human diseases are caused by nonsense mutations that introduce a premature termination codon (PTC) into the coding sequence. The PTC results in the production of a potentially harmful shortened polypeptide and activation of a nonsense-mediated decay (NMD) pathway. The NMD pathway reduces the burden of unproductive protein synthesis by lowering the level of PTC mRNA. There is an endogenous rescue mechanism that produces a full-length protein from a PTC mRNA. Nonsense suppression therapies aim to increase readthrough, suppress NMD, or are a combination of both strategies. Therefore, treatment with translational readthrough-inducing drugs (TRIDs) and NMD inhibitors may increase the effectiveness of PTC suppression. Here we discuss the mechanism of PTC readthrough and the development of novel approaches to PTC suppression. We also discuss the toxicity and bioavailability of therapeutics used to stimulate PTC readthrough.

## 1. Introduction

Protein synthesis consists of two steps, transcription, and translation, and plays a crucial role in all living organisms. Strategies for the correction of miscarried protein synthesis should be implemented before the synthesis process is complete. In eukaryotic cells, transcription and translation occur separately. Transcription followed by pre-mRNA processing takes place in the nucleolus, whereas translation takes place in the cytoplasm [1]. Mutations in the coding region of the DNA sequence are reflected in the gene transcript, which can result in the production of non-functional proteins. Single-base substitutions are termed point mutations and are classified as nonsense mutations, missense mutations, and silent mutations [2]. Silent mutations are harmless; they occur in DNA and result in a new codon that codes the same amino acid as the original codon and causes the production of an identical protein. A missense mutation is a type of deleterious mutation that can result in a codon that codes for a different amino acid than the wild type. Nonsense mutations are the most dangerous type of point mutation and occur in DNA sequences, resulting in the introduction of a premature termination codon (PTC). When the PTC comes to the A site of the 80S ribosome, the translation will be terminated, resulting in a nonfunctional or truncated protein product [3]. This nonfunctional protein can be toxic to cell function, and for this reason, mammalian cells have developed a mechanism to control the quality of mRNAs. Hence, a transcript with a PTC undergoes degradation by the nonsense-mediated mRNA decay pathway [4]. About 11% of all genetic disorders in humans are caused by nonsense mutations. [5,6]. Studies have shown that the stop codon is associated with several rare genetic diseases including cystic fibrosis (CF) [3], Duchenne muscular dystrophy (DMD) [7], spinal muscular atrophy (SMA) [8], neurofibromatosis [9], and retinitis pigmentosa [10], as well as cancer progression [11].

An important subset of genetic disorders is the frameshift or nonsense mutations, which generate PTCs; as result, the ribosome produces truncated protein [12,13]. Several strategies have been developed to counteract the severity of nonsense-mediated decays (NMDs). One of these strategies is the use of different pharmacological agents to induce nonsense suppression or readthrough of PTCs, and in this form, the full-length functional protein is expressed. For this, the use of antibiotic aminoglycosides has been recommended but they have side effects such as toxicity. Over the past decade, the use of small molecules with nonsense-suppressing abilities has been suggested to reduce toxicity during PTC targeting for several diseases.

In this review, the mechanisms associated with the recognition of PTCs, the etiology of many commonly investigated PTC-related diseases, and the drugs involved in the investigation and treatment of PTC-mediated diseases will be discussed.

## 2. The Nonsense-Mediated mRNA Decay (NMD) Machinery

**Figure 1:**
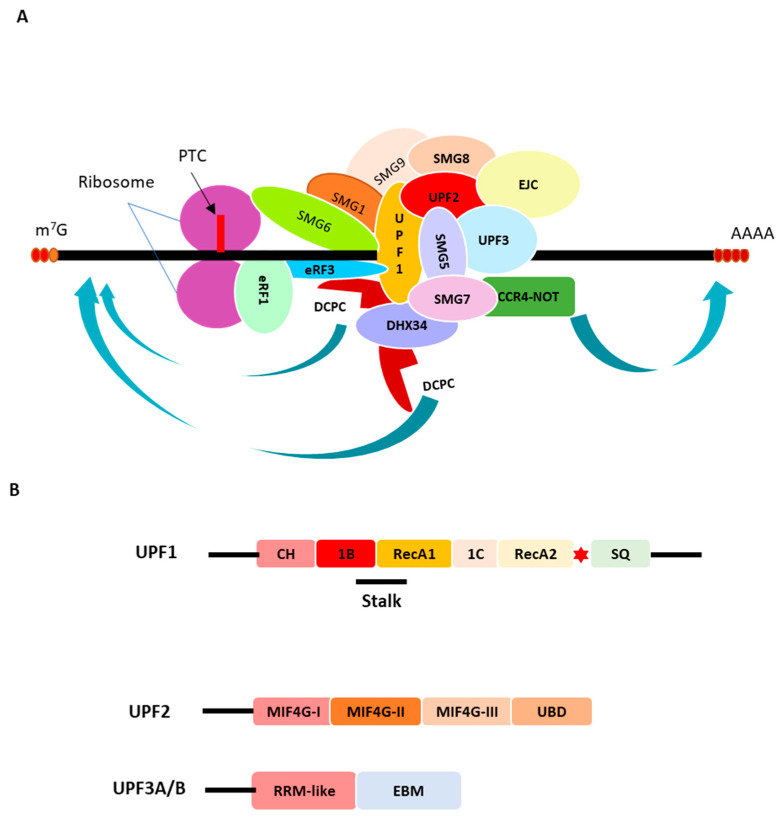
Schematic representation of the NMD factors. (**A**) The NMD reaction of the NMD factors. UPF: up-frameshift; SMG: suppressor of morphogenetic effect on genitalia; DHX34: DEAH box polypeptide 34; DCPC: decapping complex; EJC: exon junction complex; CCR4-NOT: carbon catabolite repressor protein 4 (CCR4)-NOT deadenylase complex. (**B**) The UPF protein construction. CH: cysteine–histidine-rich domain; 1B and 1C: subdomains within the helicase core; Stalk: RecA1 domains by two long “stalk” helices; RecA1 and RecA2: RecA-like domains; 1B and 1C: subdomains within the helicase core; SQ: serine–glutamine-rich domain; RRM: RNA recognition motif; EBM: exon junction motif; MIF4G: middle of 4G-like domains; UBD: UPF1-binding domain.

The NMD pathway was first elucidated using genetic screening methods in Caenorhabditis elegans and Saccharomyces cerevisiae [14,15]. Seven genes (SMG 1-7; suppressors with morphological effects on genitalia proteins 1–7) that are key components of the NMD pathway were identified in nematodes. Mutations in the SMG1, SMG2, or SMG5 genes were shown to be non-lethal, indicating that NMD is not essential for nematodes [14]. The UPF1-3 (up-frameshift) genes, which are orthologs to the SMG2, SMG3, and SMG4 genes found in C. elegans and are required for increased turnover of mRNAs containing an early stop codon, were identified in Saccharomyces Cerevisiae [15]. UPF1, UPF2, UPF3a, and UPF3b are known as NMD members, which suppress the morphological effects on the genitalia proteins (SMG1, SMG5, SMG6, SMG7, SMG8, and SMG9) and the exon junction complex (EJC) (EIF4A3, MAGOH, RBM8A, and Bartentsz (BTZ)) (see Figure 1 for the scheme) [16,17,18,19]. During translation, some EJC components are displaced by the ribosome, and this positional information is preserved by the EJC until the mRNA is translated [20,21]. During the presence of PTCs, translation is paused upstream of the EJC, and the release factors (eRF—eukaryote release factor) are likely to bind and recruit UPF1 (the RNA helicase) [22,23,24]. The eRFs can recognize the stop codon and when the mRNA with stop codons is placed into site A of the ribosome, the termination of protein synthesis occurs. eRF1 can recognize all three stop codons [25].

The SURF complex, which consists of the UPF1, SMG1, eRF1, and eRF3 proteins, is modulated by the NMD pathway. UPF3b can connect with the EJC and anchors UPF2. Thus, the SURF complex binds to the downstream PTC components, UPF2, UPF3b, and the EJC, forming the degradation-inducing complex (DECID) [26]. The SURF complex triggers the phosphorylation of UPF1 through SMG1. The multiprotein complex of SMG5, SMG6, SMG7, and protein phosphatase 2A is required for the phosphorylation of UPF1 [27].

The UPF1/SMG2 protein, which is also known as an ATP-dependent RNA helicase, undergoes cycles of phosphorylation that are required for NMD progression. UPF1 is involved in the translation termination process when the EJC lies downstream of the termination event. When UPF1 lies downstream of the EJC termination event, it is involved in the translation termination process. UPF1 undergoes different conformation changes by binding to the UPF2 protein that triggers and promotes RNA-helicase activity [28,29,30]. DEAH box polypeptide 34 (DHX34; Figure 1A) [31,32], an RNA helicase of the DEAH box family, is involved in the activation of UPF1 phosphorylation and mediates a change in interaction patterns within the NMD [33,34,35].

### The Codon Effects

The genetic code is degenerate, meaning that the use of three-letter codons in mRNA (which are decoded in the A site of the ribosome and interact with anticodons or its cognate tRNAs) allows for 64 codons to encode only 20 amino acids and signal for stop codons during translation. This leads to degenerated codons, where multiple codons code single amino acids. These codons are well known from cell organelles called ribosomes [36,37]. For many years, it was thought that different codons were translated at different speeds. The rate of translation elongation depends on the pool of tRNAs because the ribosomes have been shown to wait longer for cognate tRNAs to enter the ribosome A site. It has been shown that in a cell-free translation system, a codon-optimized mRNA (1.6kb) completes translation 1.5 min faster than an unoptimized control [38]. In a recent study, a fluorescent-tagged intracellular antibody co-expressed with the epitope protein was used to monitor the translation of single mRNAs in mammalian cells in real time. The analysis revealed that the translation of codon-optimized mRNAs occurred at a rate of 4.9 codons per second, compared to a rate of 3.1 codons per second for the non-codon-optimized mRNAs, which was an increase of 58%. These findings were reviewed in [36].

The elongation speed in the transcript is not uniform. Ribosome interruption is the subject of mRNA surveillance [39,40,41]. New assay methods have been developed to take advantage of ribosome profiling to analyze the phenomena associated with ribosome interruption. For example, Diament and colleagues developed new methods to analyze ribosome sequencing and found that in yeast, every five ribosomes were interrupted [42]. The sequence analysis in yeast showed that the sequences CGA-CCG and CGA-CGA, as well as the poly(A) tract, promote ribosome interruption [40,41,43]. A recent study by Han and colleagues showed that ribosome dimers can occur at Pro-Pro/Gly/Asp, Arg-X-Lys E-P-A sites, stop-codon sequences, and 3’UTRs in both human and zebrafish cells [44]. However, another study by Meydan and colleagues showed that the recognition of stalled ribosomes does not always result in nascent peptides in yeast [45]. 

In the last decade, many biopharmaceutical companies have made impressive efforts in the field of therapy and vaccine strategies. DNA and RNA technologies based on nucleic acids are some of the latest extreme approaches to the treatment and prevention of numerous diseases. Specifically, in the last decade, mRNA has become a therapeutic solution. mRNAs have great potential for clinical use as a vaccine against infectious diseases, cancer, rare genetic diseases, and protein replacement therapies [46,47,48,49]. 

Nonsense suppression arises from interactions with near-cognate aminoacyl-tRNA (aa-tRNA) or suppressor tRNAs inserting an amino acid in place of a stop codon. Each stop codon has a different percentage of termination fidelity, UGA (opal) > UAG (amber) > UAA (Roche) [50,51]. The application of readthrough reagents to treat the retinitis pigmentosa GTPase regulator (RPGR) gene, as well as other symptoms, may be of clinical relevance. The most common type of PTC found in exon open reading frame 15 (ORF15) is UAG (48%), followed by UAA (33%) and UGA (19%) (HGMD; http:www.hgmd.cf.ac.uk/ac/index.php).

The most common amino acid used for decoding UAA readthrough is glutamine, whereas for UAG, it is either glutamine or tryptophan. Tryptophan is used to decode the stop codon UGA [50]. A study on the suppression of the stop codon identified the nucleotide downstream of the stop codon (+4 with the first nucleotide of the termination codon as +1) as having a strong influence on the strength termination signal. The effect of the +4 position on termination efficiency was shown to be G > U, A > C for the stop codons UAA and UGA, whereas for the stop codon UAG it was U, A > C > G [52]. The order of the +4 nucleotide, which had a significant effect on the reading activity in the mammalian system proposed by Howard and colleagues, was C > U > A ≥ G, which is associated with the effectiveness of translation termination [53]. Several studies in mammalian systems report on rank order for efficiency in readthrough at the +4 nucleotide. It was suggested that the base 3′ for UAG is C > G = U > A [54], whereas the order is U > C, G > A for UAGN; C > U > G > A for UAAN; and C > A, G > U for UGAN [55]. In vitro, the suggested order is C ≈ U >> G ≈ A for UGAN [56]. A nonsense mutation (or PTC) resulting in UAG (40.4%), UGA (38.5%), or UAA (21.1%) can arise from 23 different nucleotide substitutions. It has previously been shown that CGA to TGA (21%) and CAG to TAG (19%) are the most frequent substitutions. PTCs can arise from errors during transcription, mRNA processing, or during non-faulty mRNA metabolism [57].

## 3. Nonsense-Mediated mRNA Decay (NMD) Inhibition by Drugs

Processing of mRNAs is a very important and highly regulated process in mammalian cells through different processes such as alternative splicing, translation, localization of mRNA, and NMD [58]. NMD is a highly conserved pathway for the surveillance and degradation of abnormal mRNAs, which are identified based on the presence of premature termination codons [59]. This mechanism means that it is possible that the faulty mRNAs do not fully translate into non-functional proteins. 

In 2001, the use of drugs or siRNAs for the inhibition of NMD in the treatment of cancer patients was proposed [60,61,62]. The stability of mRNAs is associated with the prevalence of malignancy; three cellular pathways, Ras/MAPK, JAK/STAT3, and NF-kB, have been associated with the role of RBM8A protein in liver cancer. [60,63]. The depletion of RBM8A was shown to inhibit these three pathways. 

Other conserved signaling regulatory pathways can modulate NMD efficacy. miR-128 is shown to be involved in the inhibition of NMD by targeting UPF1 during neuronal development [64]. Actin function is interrupted by different inhibitors (polymerization inhibitors), which can block NMD and activate PTC readthrough. PTC readthrough requires UPF proteins, which are present in P-bodies. This suggests that the differences that occur in the cytoskeleton are involved in various stages of NMD [65]. mRNAs can escape from NMD when eRF3a (eukaryotic release factor 3a) is located close to the upstream termination codon. eRF3a inhibits NMD in a poly-A-binding protein C1 (PABPC1)-dependent manner, whereas eIF4G inhibits NMD in a PABC1-independent manner [66]. A cis-element located in the 3′ UTR downstream of the codon has been shown to have the ability to inhibit NMD [67].

Several other NMD inhibitors have been used as potential therapeutic NMD inhibitors. For example, aminoglycoside compounds with low-molecular weights of 300–600 Da are commonly used as antibiotics to treat Gram-negative bacterial infections [68]. Aminoglycosides can bind at a specific site on the ribosome to control the codon–anticodon interaction between mRNA and tRNA. [69]. The first aminoglycoside used in the clinical treatment of Gram-negative bacteria infection was streptomycin. [70]. Neomycin, kanamycin, netilmicin, amikacin, isepamycin, netilmicin, arbecaci, gentamicin, gentamicin, tobramycin, paromomycin, and geneticin are other aminoglycosides that have been used to test their readthrough effects [71]. According to their chemical structure, aminoglycosides can be divided into two classes: (1) 4,6-substituted 2-deoxystreptamine ring-containing aminoglycosides, and (2) 4,5-substituted-2-ring dexystrepamine [55]. Aminoglycosides have a complex interaction with 80S ribosomes in eukaryotes. They can inhibit subunit movement and thus affect protein synthesis [72]. At low doses, these aminoglycosides can strongly bind to the decoding center and induce translation misreading [73]; at high doses, they can inhibit translation by interfering in protein synthesis [74,75]. Aminoglycosides have a higher affinity for binding to RNA duplexes [76]. For example, neomycin has been shown to increase the thermal stability of triplex DNA but has no effect on the stability of B-DNA duplexes [77]. Neomycin is also involved in the formation of a triplex DNA:RNA hybrid [78]. Another report showed that using antibiotics in OTC (over-the-counter) medicines presents a risk for cross-resistance in a number of bacterial respiratory pathogens [79]. Gentamicin has the ability to induce the readthrough of the nonsense codon in yeast and eukaryote cells, showing good potential for the treatment of genetic diseases. This was demonstrated by Burke and Mogg [80]. Tobramycin is an aminoglycoside, which, at low levels, has been shown to suppress PTC. However, gentamicin, another aminoglycoside, has been shown to be highly toxic (ototoxicity and neurotoxicity) [81]. Other aminoglycosides such as G418 and paromomycin have been shown to have the greatest stop-codon readthrough effect [82,83]. The efficiency of aminoglycosides in reading the stop codon is dependent on the efficiency of the translation termination of the stop codon in the leaking of a drug [53]. For example, Floquet and colleagues showed that the presence of gentamicin at position-1 upstream of the stop codon had a significant prognostic effect on readthrough productivity [84]. 

Cystic fibrosis (CF) was the first genetic disease associated with the potential benefits of aminoglycoside as an enhancer of PTC readthrough. Aminoglycoside has been tested as a potential suppressor of CF in vitro, in cell cultures, and in animal models. CF is caused by a mutation in the CFTR gene that leads to premature termination and a truncated protein. G418 and gentamicin were the first aminoglycosides shown to suppress nonsense mutations in the CFTR gene and increase the translation of the full-length CFTR protein [85]. The therapeutic potential of other aminoglycosides has been tested in several genetic diseases, as mentioned previously. The mechanism of G418 in translation termination has been studied and described in detail. G418 was shown to block peptide translation by binding to the 80S ribosome, inhibiting the elongation of protein synthesis [86]. The G418 mechanism to suppress PTCs has also been investigated in many other diseases. ELX-02, an alternative to gentamicin (synthetic aminoglycoside), binds to eukaryotic ribosomes and induces the readthrough of PTCs without the toxicity of the antibiotics. In particular, ELX-02 can restore the synthesis of the CFTR protein in the presence of nonsense mutations and increase the levels of mRNA [69,84]. ELX-02 has been shown to have a greater selectivity for cytoplasmic ribosomes than gentamicin. In one study, ELX-02 was administrated subcutaneously at doses of 0.3 to 7.5 mg/kg and intravenously at doses of 0.3 mg/kg. These studies confirmed that the subcutaneous administration of EXL-02 is widely used, very well tolerated, and does not show toxic effects [69]. ELX-02 is also less toxic and more efficiently promotes readthrough than gentamicin. For example, the compound NB-124 (synonym of ELX-02) can restore 7% of wild-type CFTR activity [87]. ELX-02 is currently at phase 2 of clinical trials for patients with CF who carry the G542X nonsense mutation [69,81,88]. Sharma et al. created and characterized homozygous CF rat models with the CFTR G542X nonsense mutation. Additionally, gene therapies may be a useful treatment option for CF patients carrying nonsense mutations, and the G542X rat model could serve as an important tool for future treatment testing. Multi-agent therapy to augment readthrough could be an alternative approach [89].

ELX-02-disulfate or ELX-02ds can inhibit SMG1 (part of the NMD mechanism) and correct VX-445 and VX-661 and potentially VX-770. The amount of swelling achieved by a fivefold combination of pharmacotherapies was higher than the mean swelling of three organoid cultures with the F508del/F508del mutation that were rescued by VX-809/VX-770, indicating that the level of rescue achieved is of clinical significance and comparable to that of VX-770/VX8-mediated rescue. F508del/F508del rescue in organoids is associated with substantial improvements in clinical outcomes. The data suggest that the strong pharmacological rescue of PTCs requires a combination of drugs that target RT, NMD, and protein function [90].

The modification of aminoglycosides has proven to reduce toxicity while maintaining their ability to promote readthrough [91,92]. The co-administration of poly-L-aspartic acid with gentamicin has been shown to increase the duration of CFTR function by readthrough compared to gentamicin treatment alone [93]. The small molecule SRI-37240 and its potent derivative SRI-41315 can induce a prolonged pause at the stop codon and restore CFTR expression and function by suppressing PTC-associated cystic fibrosis in primary human bronchial epithelial cells [94].

A patient diagnosed with CF cannot receive the direct benefits of a pharmacological corrector. Venturini and colleagues evaluated a combination of drugs that targets several effects of nonsense mutations G418 and ELX-02 for readthrough, VX-809 and VX-445 to promote protein maturation and function, and PTI-428 to enhance protein synthesis in patients with CFTR. The same authors indicated that the treatment of patients diagnosed with CF nonsense mutations requires a precision medical approach, including the design of a specific drug combination for each mutation [95].

To understand the function of kanamycin, paromomycin, and neomycin as potential suppressors, they were tested in the treatment of patients with PTC-associated xeroderma pigmentosum C (XPC). It has been shown that the XPC protein level is increased in these patients [96]. Paromomycin was shown to induce the readthrough of PTCs in mouse models for APC-mediated colon cancer [97]. Amikacin is a member of the kanamycin family and was shown to induce the readthrough of PTCs in the p53 gene [98,99] (Table 1).

### 3.1. Second Generation of Aminoglycosides Used for Pharmacological Therapy

According to data collected by Global Cancer Incidence, Mortality, and prevalence 2020, it is estimated that one in five people worldwide will develop cancer during their lifetime. The greatest influencing factors for this are the growth of an aging population and various socioeconomic risk factors (GLOBOCAN, 2022) [102]. Cancer treatments include surgery as an option, radiation, and chemotherapy, or a combination of these treatments. Chemotherapy consists of the administration of one or more chemicals that can damage fast-growth cells such as cancer cells. However, it has been shown that these agents can sometimes damage healthy cells and have severe toxic effects. To overcome these limitations, other therapeutic strategies have been developed that are based on the use of small molecules, gene therapies, small RNAs, and plasmids; however, strategies also have limitations due to their poor stability in vivo [103,104]. 

One of the negative effects of the use of aminoglycosides during suppression therapy is the low efficiency of the majority of aminoglycoside drugs. Many aminoglycosides and their derivatives have been shown to have toxic effects and, therefore, many strategies have been developed to avoid this toxicity. The first and second generations of these compounds are derivatives of paromomycin NB30 and NB54 [105,106,107]. These compounds are less toxic compared with gentamicin, G418, and paromomycin [106,108,109], and induced the readthrough of PTCs in Usher syndrome (US), CF, DMD, and Hurler syndrome (HS) [57]. Compounds NB74 and NB84, which were derived from G418, were effective in the readthrough of PTCs in the RTT-associated MECP2 gene [88]. Paromomycin-derived NB30 and NB54 showed up to a 15-fold reduction in toxicity [106]. TC007 is another aminoglycoside derivate of neomycin that belongs to the class of pyranmycins, which promote PTC readthrough [110]. TC007 increased and restored the level of survival motor neuron (SMN) protein in patients with smooth muscle atrophy (SMA) [110]. NB124, also known as ELX-02, was shown to induce PTC readthrough in the tumor suppressor gene p53 and APC, with a significantly higher efficiency than gentamicin, and restored the production and function of the full-length protein [81].

### 3.2. Drugs That Induce Readthrough of PTCs by Non-Aminoglycoside Compounds

Rare diseases are a challenge, as they often take some time to diagnose, and therapeutic options are uncommon. Generally, therapeutic approaches for rare diseases include treatment with antibody therapy, protein replacement, oligonucleotides, gene therapy, small molecules, and drug repurposing [111,112]. Most rare diseases are inherited in a monogenic way. Nonsense mutations, referred to as PTCs, were shown to be responsible for at least 10% of all cases of monogenetic diseases [113]. Aminoglycosides, as well as non-aminoglycosides, have been shown to induce readthrough in the highly susceptible variant COL4A5-R1563X [114].

It is very important to develop novel effective drugs for suppressing nonsense mutations. Many different libraries of small molecule candidates have been tested and this testing is ongoing. PTC124 (Ataluren or Translarna^TM^) is a 3-[5-(2-fluorophenyl)-1,2,4-oxadiazol-3-yl]-benzoic acid with the chemical formula C15H9FN2O3 and a 284.24 Da molecular weight. PTC therapies, including over 800.000 small molecules, have been tested using firefly luciferase-based readthrough reporters [115,116]. Firefly luciferase tests the ability of a compound to induce PTC readthrough in genes that contain premature termination codons (UAA, UGA, UAG). In a high-throughput screening (HTS) study of ~800,000 small molecules using firefly luciferase-based read reporters, PTC Therapeutics, Inc., found that ataluren (PTC124 or Translarna) exhibited firefly luciferase activity at very low concentrations ranging from 0.01–0.1 µM [115]. Studies performed by different research groups on genetic diseases, including DMD, CFTR, and different types of cancer, showed that PTC124 induces readthrough of a nonsense mutation [57]. Additionally, PTC124 has been shown to be well tolerated in long-term treatments [3]. The mechanism that triggers readthrough is not yet completely clear. It is thought that it may stimulate the incorporation of the closely related tRNAs into the PTCs [117]. However, it is unclear whether its behavior is similar to the aminoglycoside mechanism (by interacting with the A site in the ribosome) or if it interacts directly with the stop codon to promote reading [118,119]. Ng et al. found that ataluren can stimulate readthrough and inhibit release factors. It has been suggested that the low toxicity of ataluren aids in the development of other compounds that can inhibit termination in premature stop-codon (PSC) diseases [120].

In other studies, the action of PTC124 was shown to be controversial. Some studies reported that PTCs were not suppressed using PTC124 in vivo and in vitro [121,122]. A study performed by Krall and colleagues showed that the use of PTC124 in 293 T/17 cells that contained DNA mutations with two nonsense mutations did not yield significant results. However, if used in vivo, PTC124 was shown to produce the corresponding bmp4 alleles in Danio rerio [123]. Harmer and colleagues showed that the effect of PTC124 on the nonsense mutations R518X-KCNQ1 and Q530X-KCNQ1 caused long QT syndrome type 1 in HEK-293 cells [124]. Kosmidis and colleagues showed that PTC124 could not suppress the premature stop codons in R1638X and W153X mutations in SCN5A genes [125]. 

Negamycin or 2-(3,6-diamino-5-hydroxyhexanoyl)-methylhydrazino] acetic acid, which is a peptide-like antibiotic, was described by Hamada et al. 1970 [126]. Negamycin has been shown to bind with small and large ribosomal subunits. In small subunits, it was shown to bind to the 16S rRNA and the anti-codon loop of the A site. In prokaryotes, the 50S ribosomal subunit was shown to bind to the nascent chain in the exit tunnel and form bonds with the anticodon of the A-site tRNA [127,128]. The negamycin analog 11b was shown to be less toxic during the treatment of DMD patients [129]. Another analog, TCP-1109, was shown to be twice as potent as gentamycin in a dose-dependent manner [130]. 

Tylosin is a macrolide antibiotic composed of a 16-membered branched lactone and three deoxy-sugar residues [131]. Tylosin has been shown to bind with the E-site of the ribosome, inhibit protein synthesis, induce the readthrough of a PTC in the APC gene, and improve tumorigenic symptoms [97,101,132].

To date, only 12 compounds of low-molecular weights have been identified among the 34,000 compounds that can induce readthrough of a nonsense mutation. Of these twelve, only RTC13 and RTC14 have shown the ability to trigger PTC readout in the ATM gene [133] and restore full-length dystrophin in a mouse model of DMD [134]. RTC13 has been shown to be more effective than gentamicin in inducing readthrough in the dystrophin gene [100]. However, these compounds were unable to suppress PTC-associated hemophilia A [133]. In addition, it is not yet known how RTC13 and RTC14 bind to the ribosome and induce reading. Using the high-throughput screening (HTS) method, 2 novel compounds, GJ071 and GJ072, were found to induce readouts at all 3 stop codons identified among a collection of 36,000 small molecules [100]. Other compounds, RTC204, RTC219, NV2445, NV2907, NV2909, NV2899, and NV2913, were also identified through HTS that were shown to have PTC-readthrough properties like PTC124, gentamicin or G418 [133,135], and PTC414 [136].

Another compound is clitocin, which is isolated from fungi and was shown to have the ability to incorporate into RNA molecules during transcription. Clitocin can restore the full-length p53 protein in cell cultures and xenograft tumor mouse models with p53 mutations [137]. 2,6-diaminopurine (DAP) is another compound that is isolated from mushrooms, which can induce readthrough of the UGA stop codon in the TP53 gene in cancer cells [138].

Amlexanox is an anti-allergic and anti-inflammatory compound that was shown to induce efficient readthrough of PTCs in mRNAs expressed in human culture cells [139]. Amlexanox was also shown to induce readthrough of the COL7A1 gene in patients with recessive dystrophic epidermolysis bullosa (RDEB) [140].

Dabowski et al. tested a group of compounds with known PTC-readthrough potential (ataluren, azithromycin, tylosine, amlexanox, and TC007), collectively known as non-aminoglycosides (NAGs). They investigated the efficiency of PTC readthrough in six PTC mutations in the context of cell and cilia health in Polish patients and compared the results to previously tested aminoglycosides. The NAGs did not alter the variability of the primary nasal respiratory cells and the ciliary beat frequency was maintained, similar to that observed for gentamicin [141] (Table 2).

**Figure 2:**
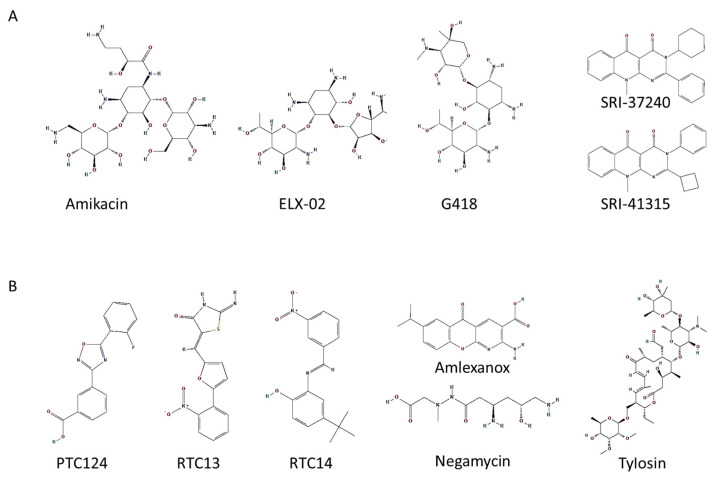
Some of the chemical structures of the compounds mentioned in Table 1 (**A**) and Table 2 (**B**). (Chemical structures modified from https://pubchem.ncbi.nlm.nih.gov/, accessed on 18 February 2023).

Figure 2 shows the chemical structures of some representative compounds mentioned in Table 1 and Table 2.

### 3.3. Combinatorial Drug Therapy

Nonsense suppression studies with aminoglycosides and non-aminoglycosides have shown promising results in the treatment of various PTC-derived genetic disorders. There were considerable differences in the therapeutic outcomes of pre-clinical and clinical studies between mice and human patients. For instance, patients with the W1282X nonsense mutation showed varying CFTR mRNA levels, which correlate with the response to gentamicin treatment [142]. These results indicate that, in some cases, nonsense suppression therapy alone may not be enough and combinatorial therapy may be required for these patients. In one such case, transcriptional and translational processes were regulated to obtain higher functional protein synthesis through a combination treatment of aminoglycosides such as gentamicin or G418, and promoter-activating agents such as ofloxacin or thioguanine [143].

Studies were conducted on another type of combination therapy to minimize the toxicity associated with aminoglycoside treatment. A study by Barton-Davis et al. showed the attenuation of gentamicin-induced toxicity through the co-administration of 2,3-dihydroxybenzoate (DHB) in mdx mice [144]. Similarly, the co-administration of polyanions such as poly-L aspartic acid (PAA) and aminoglycoside therapy weakened the nephrotoxicity and increased the intracellular retention of aminoglycosides [145]. The in vivo co-administration of PAA and gentamicin in a CF mouse model resulted in increased suppression of the CFTR-G542X nonsense mutation [93]. Likewise, the co-treatment of G418 and gentamicin synergistically increased the synthesis of methylmalonyl-CoA mutase mRNA [146].

In principle, NMD suppression increases the frequency of nonsense-containing mRNA, which can then be targeted by nonsense suppression molecules to increase full-length protein production. The level of CFTR nonsense transcripts is associated with the response of patients to gentamicin treatment [142]. The administration of gentamicin, along with UPF1 siRNA, provides a synergistic improvement in CFTR activation. This indicates that NMD regulates the level of nonsense transcripts and influences nonsense suppression therapies.

### 3.4. Challenges Associated with Drug-Stimulated Nonsense Suppression Studies

Although aminoglycosides have been used in clinical applications, they still cause oto- and nephrotoxic effects on the organism, leading to necrosis and the apoptosis of the cells. A study on hair cells showed the disarray of stereocilia, as well as apoptosis [147]. The selective toxicity of aminoglycosides was detected in the inner ear and kidneys as a result of the cellular uptake of megalin, an endocytic receptor located in the apical membrane of epithelial cells present in the proximal renal tubules and the hair cells of the inner ear [148]. The positively charged aminoglycosides interacted with phospholipids to form complexes, which aggregated in the lysosomal membranes, causing phospholipidosis and leading to nephro- and ototoxicity [149]. Furthermore, these changes led to the generation of ROS and, ultimately, apoptosis [150]. Aminoglycosides also induced oxidative damage to the mitochondrial enzymes, leading to the generation of hydroxyl radicals via the Fenton reaction and, finally, cell apoptosis [151]. In contrast, the long-term administration of gentamicin in DMD patients showed no toxicity.

Furthermore, the practical applications of these compounds may have some limitations due to cell permeability problems in reading compounds. Almost all mammalian cells can take up aminoglycosides during endocytosis or non-endocytotic pathways. However, at lower temperatures when endocytosis decreases, Texas Red (GTTR)-labeled gentamicin in the cytoplasm conducts ROS production. [152]. Other important factors are shown to be involved, including the method of administration and the choice and duration of doses. PTC-readthrough stimulation is a transient phenomenon, but for therapeutic purposes, it may be necessary to repeat drug administration, prolong treatment, and in some cases, administer components for a lifetime. The effective dose of the non-aminoglycoside compound PTC124 was studied in phase I and II clinical trials. PTC124 is rapidly absorbed after oral administration and shows a dose-proportional increase in pharmacokinetic parameters [116]. Therefore, the selection of an effective drug administration regimen is also important.

In addition, other more elusive PTC-readthrough-related problems exist, and finding ways to overcome them is difficult. The accessibility of a PTC-bearing transcript is a challenge. In the cell, mRNA availability is closely related to the efficiency of the NMD process [153]. When NMD is efficient, the level of mutant mRNA is markedly reduced, and even if potent nonsense-suppressing drugs are provided, the amount of functional protein is very low [154].

### 3.5. Development of NMD Inhibitors: New Pharmacological Perspectives

NMD is the most studied quality-control mechanism [11,16,155]. The number of drugs that have been identified that affect the first round of translation, as well as their activity, in the exo and endonucleolytic pathways is currently very large [4].

The NMD inhibitors are good candidates for future therapies. The small molecule NMDI-1 participates in the stabilization of the UPF1 protein during hyperphosphorylation and reduces the interaction between core NMD factors UPF1-SMG5 [156,157,158]. Pateamine A (Pat A) inhibits the NMD pathway through direct interaction with the core EJC protein eIF4AIII [158,159]. The dietary compound curcumin has been shown to inhibit the NMD pathway through the downregulation of the expressions of core NMD factors at the transcriptional level [160,161]. Pyrimidine derivatives can also inhibit the core elements of the NMD pathway such as SMG1 [162]. Wortmannin and caffeine are other compounds that have been identified as NMD inhibitors, which can increase the expression of the mutant collagen VI α2 subunit [161,163].

The inactivation of p53 is present in all tumors and p53 reactions are very important for cancer therapy. The most common mechanism for p53 loss in cancer cells is the expression of p53 negative regulators such as MDM2, which mediates the degradation of wild type p53 and the inactivation of mutations in the TP53 gene. NMD inhibition in cancer cells has been shown to upregulate two alternatively spliced function truncated isoforms of p53 (p53β and p53γ) and restore the p53 pathway. The inhibition of NMD has been shown to induce tumor-suppressive activities such as apoptosis, reduce cell viability, and enhance tumor radiosensitivity. NMD inhibition has also been shown to inhibit tumor growth in an MDM2-overexpression xenograft tumor model. The results of a study by Gudikote and colleagues showed that NMD inhibition is a new therapeutic strategy for restoring the function of p53-deficient tumor-bearing MDM2 overexpression [164].

Immune-checkpoint therapy (ICB) has been shown to change the clinical outcomes for many aggressive tumors. Meraviglia-Crivelli and colleagues used aptamer (AS1411) conjugation with SMG1 RNAi (AS1411-SMG1 aptamer-linked siRNA chimeras (AsiCs)) to inhibit the NMD (non-mediated decay) pathway. This aptamer was shown to bind to a large number of mouse and human tumor cell lines and induce a strong antitumor response in both local and systemic therapies, with no detected side effects [165].

It was shown that digoxin and ouabain (cardiac glycosides) increased cytoplasmic calcium and suppressed the NMD pathway [162,166]. 5-azacytidine, an analog of the naturally occurring nucleoside cytidine, has been approved as a therapeutic drug for the treatment of myelodysplastic syndrome and myeloid leukemia [167], as it can inhibit the NMD pathway by inducing the expression of the MYC gene [168].

Baradaran-Heravi et al. showed that CC-885 and CC-90009 decreased eRF3a, eRF3b, and eRF1 levels and acted synergistically with aminoglycosides to suppress NMD and increase PTC readthrough [169]. CC-90009 is less toxic than CC-885 and it has been shown to improve PTC readthrough in DMD (Duchenne muscular dystrophy), mucopolysaccharidosis type I-Hurler, late infantile neuronal ceroid lipofuscinosis, and JEB (junctional epidermolysis bullosis) patient-derived cells with nonsense mutations in the IDUA, TPP1, DMD, and COL17A1 genes. A combination of CC-90009 and aminoglycosides such as gentamicin or ELX-02 may have the potential for PTC readthrough therapy [169].

The small molecule AC1903, also known as A498, has been shown to inhibit several TRPC channels, including endogenous TRPC1:C4 channels, TRPC3, TRPC4, TRPC5, TRPC6, TRPC4-C1, and TRPC5-C1 in A498 kidney cancer cells. Moreover, AC1903 was found to inhibit TRPV4 channels but had minimal effect on TRPV1 and no effect on the non-selective cation channel PIEZO1 [170] (Table 3). Huang et al. developed antisense oligonucleotides (ASOs) that specifically block the mRNAs of core NMD factors, including the UPF and SMG proteins, to evaluate the efficacy and safety of NMD inhibition. This study discovered that among the key NMD factors targeted, ASO-mediated Upf3b depletion essentially suppressed NMD on some disease-causing mRNAs and had minimal effect on overall transcription. These results suggest that targeting UPF3B with ASOs may be a possible approach to NMD inhibition in the treatment of nonsense mutation-induced human diseases [171].

## 4. Conclusions and Discussion

The regulation of transcriptome homeostasis is achieved through nonsense-mediated mRNA decay, which plays a pivotal role in the recognition of PTCs in the transcript. NMD is involved in other processes in the organism such as development, the response to DNA damage, the cell cycle, and the immune system. Disruptions to this pathway lead to pathologies such as neurological disorders, immune diseases, and cancer. Studies have shown that the relationship between NMD and cancer is very complex. Under normal conditions, NMD has a protective role in the cell because it protects the cell from the dominant negative protein product and can downregulate the expression of many important factors that may affect tumor cells. There is increasing evidence to show that tumor cells may exploit the function of NMD to promote the onset and progression of certain diseases. The role of NMD function in cancer can be seen as a double-sided coin, as it can act as a tumor suppressor and promoter pathway depending on the genetic context.

The development of new strategies, such as the use of non-aminoglycosides, aminoglycosides, and their derivatives, could reduce the toxicity associated with long-term therapies or the use of gene editing as a suppression therapy. However, all these therapeutic approaches need further investigation and testing before they can be used in the treatment of PTC-related diseases. Developing drug combination therapy strategies is important for suppressing NMD and more research is needed. Future cancer therapies could benefit from current research aimed at developing pharmacological compounds and different strategies capable of inhibiting the negative effects of NMD on nonsense-mediated decay readthrough.

## 5. Future Directions

The nonsense codon is generated by a nonsense mutation, which significantly contributes to the large spectrum of many inherited diseases such as cystic fibrosis, DMD (Duchenne muscular dystrophy), hemophilia, spinal muscular atrophy, and many types of cancer. The presence of the nonsense mutation results in premature termination that prevents full-length protein synthesis and causes aberrant gene expression. Suppression therapy is a strategy for the treatment of the above-mentioned diseases caused by nonsense mutations. This therapy employs various pharmacological agents that can suppress the translation termination of in-frame premature termination codons (PTCs) and restore full-length protein synthesis. To date, several small molecules have been reported, which have been shown to enhance sequence-specific repair approaches that are safe and compliant with personalized medicine. The key challenges include identifying the various processes and crosstalk between the different types of machinery involved in premature termination rather than normal termination. Moreover, a detailed understanding of the mechanisms of action of the aminoglycoside and non-aminoglycoside compounds is needed. Several conventional and new technologies are being employed, with many already tested or set to be tested in clinical trials as potential therapies for nonsense mutations, although some of them may have limitations and challenges that need to be addressed.

## Figures and Tables

**Table 1:** Drugs that can suppress and restore gene mutations in different diseases.

Drugs	Gene	References
G418	CFTR	[85]
Gentamicin	CFTR	[85]
ELX-02	CFTR	[69,84]
NB-124	CFTR	[87,89]
poly-L-aspartic acid with gentamicin	CFTR	[100]
SRI-37240 and its potent derivate SRI-41315	CFTR	[101]
Elexacaftor	CFTR	[95]
Kanamycin, paromomycin and neomycin	xeroderma pigmentosum C (XPC)	[96]
Paromomycin	APC-mediated colon cancer	[97]
Amikacin	p53 gene	[98,99]

**Table 2:** Non-aminoglycoside drugs that induce readthrough.

Drugs	Diseases/Gene	References
PTC124	QT syndrome type 1	[124]
Tylosin	APC gene	[97,101,132]
Negamycin	DMD patients	[129]
readthrough compound RTC13 and RTC14	ATM gene	[93,133,134]
Clitocin	p53 protein	[137]
2,6-diaminopurine (DAP)	TP53 gene in cancer cells	[138]
Amlexanox	COL7A1 gene in patients with recessive dystrophic epidermolysis bullosa (RDEB)	[140]

**Table 3:** Different NMD inhibitors.

Drug	Gene Inhibition	References
NMD-1	Stabilization of UPF1 protein during hyperphosphorylation	[156,157,158]
Pateamine A (Pat A)	Inhibited NMD pathway	[158,159]
Curcumin	Inhibited NMD pathway	[160,161]
Pyrimidine derivate	Inhibited SMG1	[162]
Wortmanin and caffeine	Blocked NMD pathway	[161,163]
Digoxin and ouabain	Repressed NMD pathway	[163,166]
AC1903	Inhibited multiple TRPC channels in renal cancer	[170]

## Data Availability

Not applicable.
